# Pomelo Peel and Soybean Meal Fermented Compound as Feedstuff for Large Yellow Croaker (*Larimichthys crocea*): A Study on Growth and Intestinal Health

**DOI:** 10.1155/anu/6556868

**Published:** 2025-07-11

**Authors:** Zi-Yan Liu, Yu-Hang Hong, Yi-Ling Li, Guo-He Cai, Pan Wang, Chuang-Zhong Zhu, Kang-Le Lu, Hong-Ling Yang, Yun-Zhang Sun

**Affiliations:** ^1^State Key Laboratory of Mariculture Breeding, Fisheries College of Jimei University, Xiamen 361021, China; ^2^School of Life Sciences, Sun Yat-Sen University, Guangzhou 510275, China; ^3^Xiamen Key Laboratory for Feed Quality Testing and Safety Evaluation, Fisheries College of Jimei University, Xiamen 361021, China; ^4^Key Laboratory of Aquatic Functional Feed and Environmental Regulation of Fujian Province, Fujian DBN Aquatic Sci. & Tech. Co., Ltd., Zhangzhou 363500, China; ^5^The Key Laboratory of Healthy Mariculture for the East China Sea, Ministry of Agriculture, Fisheries College of Jimei University, Xiamen 361021, China

**Keywords:** feedstuff, growth, inflammation and microbiota, intestinal histology, *Larimichthys crocea*, pomelo peel

## Abstract

Pomelo peel is a main by-product of pomelo consumption, while most of the by-products are discarded as wastes with environmental threats. Indeed, it contains many nutrients and bioactive ingredients, which makes it a promising source of aquatic feedstuff. In this study, a 56-day feeding trial was set out to assess the dietary application of pomelo peel and soybean meal (SBM) (1:4 ratio) fermented compound (PSFC) in large yellow croaker (*Larimichthys crocea*). Results indicated that dietary PSFC improved weight gain rate (WGR) and specific growth rate (SGR), but did not affect feed utilization, body shape parameters and survival rate (SR) of croakers. Moreover, PSFC significantly decreased serum diamine oxidase (DAO) activity and *D*-lactate concentration, increased muscle thickness and villus height, as well as upregulated *occludin*, *claudin-11*, *ZO-1* and *JAM* mRNA levels. The inhibition of proinflammatory *IL-1*β and promotion of anti-inflammatory *IL-4/13b* and *IL-10* expressions were documented in croakers fed with PSFC. Dietary PSFC upregulated intestinal *TLR1*, *TLR2A*, and *TLR2B* expression, which had significantly positive correlations with improved inflammatory status. Furthermore, PSFC application caused clear alterations in the intestinal microbiota of croakers, which characterized with decreased relative abundances of Verrucomicrobiota and Acidobacteriota and increased relative abundances of *unclassified_Muribaculaceae* and *Akkermansia*. Overall, PSFC can significantly improve growth and intestinal health of *L. crocea*, and showed potential as protein source on *L. crocea* culture. This work offers a novel insight into the development of fermented protein sources for aquafeeds using pomelo peel and SBM as fermentation ingredients.

## 1. Introduction

Pomelo (*Citrus maxima*) is the largest specie of citrus fruit, and its peel (~30% – 50% of the fresh fruit weight) is a well-known agricultural residue [1]. At a rough estimate, the global production of pomelo peel has reached 2.8 – 4.7 million tonnes in 2018 [2]. Due to the higher water content (~77% – 80%) of pomelo peel, the decay easily occurred during storage. As a result, improperly disposed pomelo peels caused serious pollution and environmental issues. On the other hand, pomelo peel is rich in various high-value functional components, such as naringin, pectin, essential oils, and so on [2]. These major compounds have presented multiple bioactive functions, including antimicrobial and antioxidant activities, metabolic modulation, growth promotion, etc. [3, 4]. Pomelo peels have been used as the raw materials in active carbons [5], extraction of functional ingredients (essential oils, pectin, polyphenols, etc.) [2], and animal feeds [6]. It has been reported that freeze-dried citrange peels [7] and *Cenchrus ciliaris* peel wastes [8] can be potential feedstuffs for ruminants. Additionally, fermented pomelo peel and soybean meal (SBM) mixture could improve immune response and intestinal health in largemouth bass (*Micropterus salmoides*) [9]. The high-value utilization of pomelo peels can result in the alleviation of its potential pollution and environmental issues, as well as achieve considerable economic benefits.

During the last decade, there has been a dramatic growing trend in the aquaculture industry, which played an increasingly vital role in maintaining food security and human nutrition. In the aquaculture industry, the exploitation of novel protein sources is an important topic since fishmeal, a traditional protein source of aquatic feeds, is in limited supply and has a high price [10, 11]. SBM and fermented SBM has been wildly used in aquatic feeds [11–13]. However, their applications with a high percentage will result in a series of detrimental impacts in aquatic animals. For example, high percentage of dietary SBM [14, 15] or soybean glycinin [16] could suppress growth, disrupt intestinal morphology, induce enteritis and/or microbial dysbiosis in large yellow croaker (*Larimichthys crocea*). It has been reported that dietary raw or *Enterococcus faecium* fermented SBM suppressed growth, antioxidant and nonspecific response, and caused intestinal inflammation in turbot (*Scophthalmus maximus* L.) [17]. Similarly, a previous study found that the growth, digestive enzymes activities, systemic immunity, and intestinal health were negatively changed at higher substitution levels of fishmeal with *Bacillus pumillus* SE5 fermented SBM [13]. There is an urgent need to develop a safe and effective biotechnological strategy to address the intrinsic challenges of SBM (e.g., antinutritional factors, allergenicity) that hinder its application in aquaculture feeds. Interestingly, several studies have shown that dietary pomelo peel extracts could effectively improve growth, intestinal barrier functions, and mitigate metabolic disorders in mice [3] and Holstein bulls [18]. However, to the best of our knowledge, there is no report about the application of pomelo peel in aquatic feeds. Herein, pomelo peel and SBM fermented compound (PSFC) was developed as a novel protein source of aquatic feeds

*L. crocea* is a species with high market and nutritional values, and its production approached 280,997 tons in China in 2023. In this study, the effects of dietary PSFC in *L. crocea* were investigated from the perspective of growth performance, intestinal histology, inflammation, and microbiota. The results of this study will provide a novel insight into the development of effective protein sources for aquafeeds and high-value utilization of residual wastes in the citrus processing chain.

## 2. Materials and Methods

All animal studies were approved by the Animal Care and Use Committee of Jimei University, in keeping with Chinese ethical Guidelines for Experimental Animals.

### 2.1. Preparation of Experimental Diets

*B. pumilus* SE5 (isolated from the intestine of *Epinephelus coioides*, GenBank no. EU520331), *Saccharomyces cerevisiae* Sa (isolated from Daqu, GenBank no. OK493565) and *Lactococcus lactis* 17 (isolated from the intestine of *Amphiprion ocellaris*, GenBank no. OL631477) were employed during fermentation in this study. This is because these three strains demonstrated excellent fermentation performance in previous fermentation trials conducted in our laboratory, such as significantly reducing antinutritional factors and enhancing nutritional components [19]. *B. pumilus* SE5, *S. cerevisiae* Sa, *L. lactis* 17 were respectively cultured in 2216E liquid medium (HB0132-1, Hope Bio-Technology Co., Ltd., China, Qingdao, China), YPD broth (Y0002, Beijing LABLEAD, Inc., Beijing, China), and MRS broth (LM1175B, Beijing LABLEAD, Inc., Beijing, China), and then kept at 30°C overnight. The bacterial liquids were centrifuged (4°C, 3000 × *g*, 10 min). Subsequently, the bacterial pellet was washed three times, and resuspended to an appropriate concentration in sterile PBS for subsequent fermentation. The viable counts of *B. pumilus* SE5, *S. cerevisiae* Sa, *L. lactis* 17 for fermentation were respectively 6.0 × 10^5^ CFU/g, 7.0 × 10^5^ CFU/g, and 2.25 × 10^4^ CFU/g. The PSFC was produced as follow: Pomelo peel and SBM were ground and mixed thoroughly at a ratio of 1:4 (*w:w*). The mixture was inoculated with *B. pumilus* SE5, *S. cerevisiae* Sa, and *L. lactis* 17, mixed and fermented in a thermostatic chamber at 30°C for 48 h. After fermentation, the PSFC was dried to constant weight at 40°C, grounded in fine particle size and stored at 4°C for further utilization.

The formulation and all compositions of experiment diets are displayed in Table 1. Two diets with isonitrogenous (~ 44% crude protein) and isolipidic (~10% crude lipid) were prepared containing SBM or PSFC, respectively. All dry ingredients were ground to pass a 60-mesh screen, and mixed with oil and water. The resulted stiff doughs were used to prepared expanded pellet diets (2.5 mm) by a pellet-making machine (CD4XITS extruder, South China University of Technology, Guangzhou, China).

### 2.2. Feeding Trial and Sample Collection

*L. crocea* were obtained from a fishery in Ningde, Fujian Province. After the acclimation period (14 days), fish were categorized into control and PSFC groups which were respectively fed with control and PSFC diet for 56 days. Each group was randomly assigned four replicate net cages (1.5 × 1.5 × 2.5 m^3^) with 160 fish per net cage. The fish initial average weight is approximately 19.5 g. Fish were hand-fed to apparent satiation twice a day at 8:00 and 18:00. During the acclimation and experimental period, feeding and health status were documented and water quality conditions were monitored daily (water temperature: 23.5 ± 3°C; salinity: 29%–33‰ and dissolved oxygen: ≥6 mg/L). The breeding, adaptation, and experimental location is the Sandu Bay aquaculture zone in Fujian, China.

After feeding trial, fish were anesthetized with MS222 and each fish was individually weighed (30 fish per net cage, thus total 120 fish per group). Then, tail vein blood and intestinal tissues were collected. The serum samples were separated from the tail vein blood by centrifugation. A portion of intestinal samples were immediately put into liquid nitrogen, then preserved at −80°C for further analysis. The residual intestines were soaked in Bouin's fixative solution. After removing the intestinal contents with sterile instruments, the intestinal lumen was rinsed with sterile phosphate buffered saline. The intestines of three fish in each tank were randomly taken as a sample, and four samples per group were immediately transported into liquid nitrogen and stored at −80°C until microbial analysis. The middle segment of the intestine was selected for histology analysis.

The survival rate (SR) and growth performance, including final body weight (FBW), weight gain rate (WGR), special growth ratio (SGR), feeding rate (FR), feed coefficient ratio (FCR), condition factor (CF), viscera index (VSI) and hepatosomatic index (HSI), were calculated using the recorded data and previously published formulas [20].

1. Final body weight,  FBW(g)= total final body weight/final fish number.
2. Weight gain rate,  WGR (%) = 100  × (final body weight − initial body weight)/ initial body weight;
3. Specific growth rate,  SGR(%/d)=100 × (ln [final body weight] − ln[initial body weight])/days;
4. Feed conversion rate,  FCR= total amount of the feed consumed/(final body weight − initial body weight);
5. Feed rate,  FR(%/d)=100 × total amount of the feed consumed/([initial body weight+final body weight]/2)/days;
6. Condition factor,   CF(%)=100 × body weight/body length^3^;
7. Viscerosomatic index,  VSI(%)=100 × (visceral weight/body weight);
8. Hepatosomatic index,  HSI(%)=100 × (hepatopancreas weight/body weight);
9. Survival rate,  SR(%)=100 × final fish number/initial fish number.

### 2.3. Intestinal Histology

For each experimental group, intestinal tissues were collected from three fish per cage, with a total of 12 samples per group statistically analyzed. After fixation, the intestinal samples were dehydrated and embedded into paraffin. Then, 6 μm-thick sections were cut from paraffin blocks. Subsequently, the sections were stained with hematoxylin and eosin. Finally, the intestinal histology and morphological parameters, including muscle thickness, villus height, and villus width, were recorded by a Nikon digital sight DS-FI2 system [10].

For assessing intestinal permeability, serum diamine oxidase (DAO) activity, and *D*-lactic content were detected using commercially available kits (Beijing Solarbio Science & Technology Co., Ltd.).

### 2.4. Gene Expression

The total RNA were extracted from the intestine samples using Total RNA Extraction Kit (AKNA001-1, Beijing Boxbio Science & Technology Co., Ltd., Beijing, China), and the RNA were reversely transcribed and synthesized to cDNA. Then, real-time quantitative PCR were conducted to determine the relative levels of target genes by 2 × SYBR Green qPCR Mix (Shandong Sparkjade Biotechnology Co., Ltd.). The relative expression of target genes were calculated by the 2^−ΔΔCt^ method, using β-actin as a housekeeping gene. Table S1 presented the specific primer sequences in this study.

### 2.5. Intestinal Microbiota

The total bacterial DNA of intestinal samples (four samples per group thus total 16 samples in this study) were extracted using QIAamp DNA Stool Mini Kit (QIAGEN, Germany) and amplify 16S rRNA V3-V4 region. PCR amplification products were sequenced with paired-end, and a library was constructed by mixing in proportion according to the sequencing requirements. After concatenating, screening, quality control, and filtering the dual end sequence data, QIIME2 2020.6 software was used to cluster the sequences at 97% similarity, resulting in operational taxonomic units (OTUs). Furthermore, the microbial analysis was performed on the BMKCloud platform. Raw tags were filtered using Trimmomatic (v0.33, http://www.usadellab.org/cms/?page=trimmomatic) and UCHIME (v4.2, http://drive5.com/uchime) in order to remove the low-quality sequences and chimeras. Then, sequences with ≥97% similarity were assigned to the same OTUs using UCLUST in QIIME V1.8.0 package. A representative sequence for each OTU was annotated by searching the SILVA database (http://www.arb-silva.de/). Beta diversity analysis is used to analyze changes in species composition across these three groups. Species diversity matrices are presented based on binary jaccard. Principal component analysis (PCA), principal coordinates analysis (PCoA), nonmetric multidimensional scaling (NMDS), partial least squares discrimination analysis (PLS-DA) are performed on the BMKCloud platform.

### 2.6. Statistical Analysis

All data were evaluated for homogeneity of variance by Bartlett test and normality using the Shapiro–Wilk test. Then, data between control and PSFC groups were compared with a Student's *t*-test using the Graphpad prism 8. A *p*-value ≤ 0.05 was considered statistically significant, while a *p*-value between 0.05 and 0.1 was considered as a trend. The results are displayed as the mean ± standard error of the mean (SEM).

## 3. Results

### 3.1. Growth Performance and SR

No notable differences in FBW, FCR, CF, VSI, HSI, and SR were found between the control and PSFC groups (*p* > 0.05, Table 2). However, dietary PSFC significantly enhanced WGR and SGR compared to the control group (*p* < 0.05, Table 2).

### 3.2. Intestinal Histology

**Figure 1:**
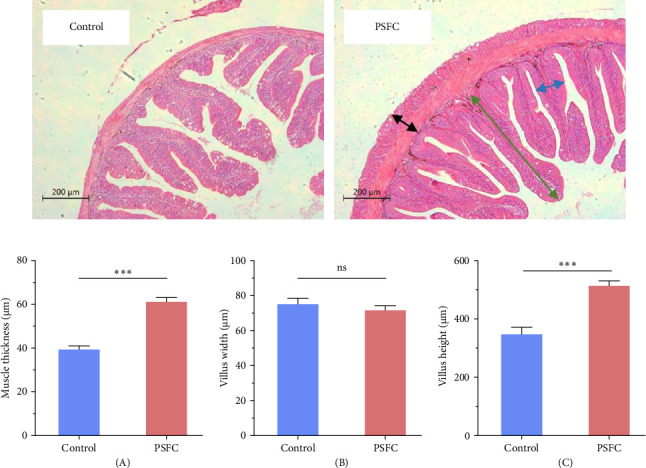
Representative histological sections showing the intestine of large yellow croaker (*L. crocea*). (A) Muscle thickness, (B) Villus width, and (C) Villus height (scale bars, 200 μm). Values are presented as mean ± SE, *n* = 4 (*⁣*^ns^*p* > 0.05, *⁣*^*∗*^*p* ≤ 0.05,  ^*∗*^^*∗*^*p* ≤ 0.01,  ^*∗*^^*∗*^*p* ≤ 0.001). Black arrow represents muscle thickness; blue arrow represents villus width; green arrow represents villus height.

Figure 1 displayed intestinal histology of *L. crocea* fed with control and PSFC diets. In control and PSFC groups, the overall structure was in good shape, and no obvious injury and inflammatory cell infiltration. Moreover, there was no significant difference between control and PSFC groups, in terms of villus width (*p* > 0.05, Figure 1B). Of note, *L. crocea* fed with PSFC diet showed significantly increased intestinal muscle thickness (Figure 1A) and villus height (Figure 1C) compared to fish fed with control diet (*p* < 0.001).

**Figure 2:**
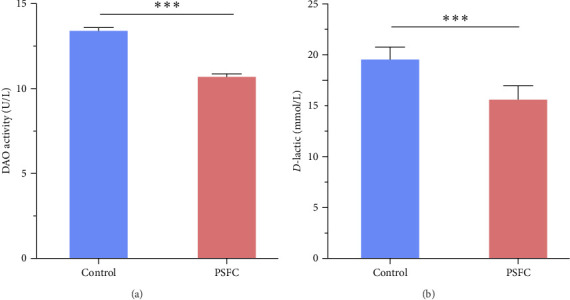
Serum *D*-lactic concentration (A) and DAO activity (B) of large yellow croaker (*L. crocea*). Values are presented as mean ± SE, *n* = 4 (*⁣*^ns^*p* > 0.05, *⁣*^*∗*^*p* ≤ 0.05,  ^*∗*^^*∗*^*p* ≤ 0.01,  ^*∗*^^*∗*^*p* ≤ 0.001).

Intestinal permeability, characterized by serum *D*-lactic concentration and DAO activity, was determined and presented in Figure 2. Significantly decreased serum *D*-lactic concentration and DAO activity were recorded in PSFC group compared to those in the control group (*p* < 0.001).

**Figure 3:**
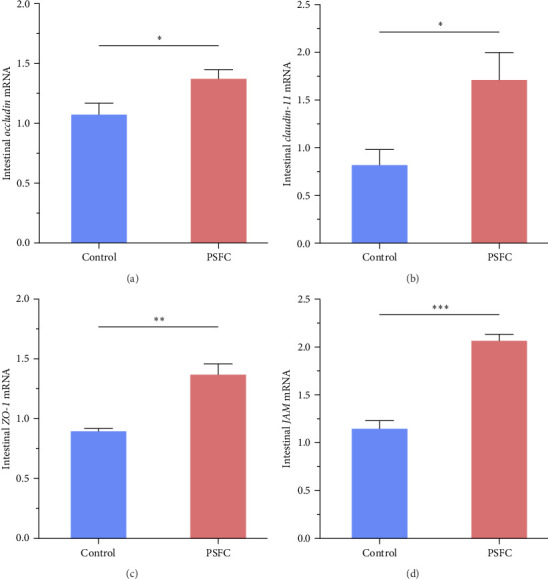
The mRNA levels of *occludin* (A), *claudin-11* (B), *ZO-1* (C) and *JAM* (D) in the intestines of large yellow croaker (*L. crocea*). Values are presented as mean ± SE, *n* = 4 (*⁣*^ns^*p* > 0.05, *⁣*^*∗*^*p* ≤ 0.05,  ^*∗*^^*∗*^*p* ≤ 0.01,  ^*∗*^^*∗*^*p* ≤ 0.001).

Significantly upregulated *occludin* (*p* < 0.05, Figure 3A), *claudin-11* (*p* < 0.05, Figure 3B), *ZO-1* (*p* < 0.01, Figure 3C) and *JAM* (*p* < 0.001, Figure 3D) mRNA levels were observed in PSFC group compared to those in the control group.

**Figure 4:**
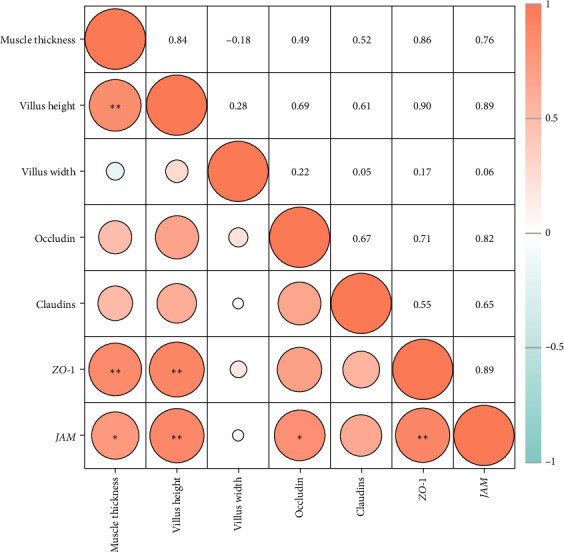
Correlations between morphometric parameters and tight junction-related genes expression in the intestines of large yellow croaker (*L. crocea*). There were significantly positive correlations between muscle thickness and villus height, as well as *JAM* and *occludin*, *claudin-11*mRNA levels. In addition, intestinal histology had positive correlations with tight junction related genes expressions; furthermore, muscle thickness and villus height had significantly positive correlations with *ZO-1* and *JAM* mRNA levels (*⁣*^ns^*p* > 0.05, *⁣*^*∗*^*p* ≤ 0.05,  ^*∗*^^*∗*^*p* ≤ 0.01,  ^*∗*^^*∗*^*p* ≤ 0.001).

As shown in Figure 4, the correlation heat map showed that there were significantly positive correlations between muscle thickness and villus height (*r* = 0.84, *p* < 0.01), as well as *JAM* and *occludin* (*r* = 0.82, *p* < 0.05), *claudin-11* (*r* = 0.89, *p* < 0.01) mRNA levels. In addition, intestinal histology had positive correlations with tight junction related genes expressions; furthermore, muscle thickness and villus height had significantly positive correlations with *ZO-1* and *JAM* mRNA levels (*p* < 0.05).

### 3.3. Intestinal Inflammatory Genes

**Figure 5:**
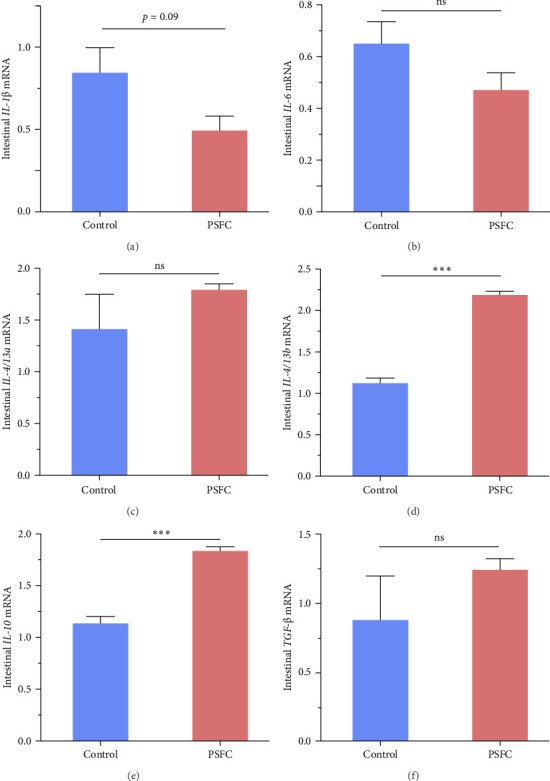
The mRNA levels of *IL-1β* (A), IL-*6* (B), *IL-4/13a* (C), *IL-4/13b* (D), *IL-10* (E), and *TGF*-β (F) in the intestines of large yellow croaker (*L. crocea*). Values are presented as mean ± SE, *n* = 4 (*⁣*^ns^*p* > 0.05, *⁣*^*∗*^*p* ≤ 0.05,  ^*∗*^^*∗*^*p* ≤ 0.01,  ^*∗*^^*∗*^*p* ≤ 0.001).

Intestinal proinflammatory *IL-1*β mRNA shows a tendency to be suppressed in PSFC group compared to control group (*p*=0.09, Figure 5A). No significant differences on proinflammatory *IL-6* (Figure 5B), anti-inflammatory *IL-4/13a* (Figure 5C) and *TGF*-β (Figure 5F) mRNA expressions were found between control and PSFC groups (*p* > 0.05). Compared to the control group, anti-inflammatory *IL-4/13b* (Figure 5D) and *IL-10* (Figure 5E) mRNA expressions were significantly upregulated in PSFC group (*p* < 0.001).

**Figure 6:**
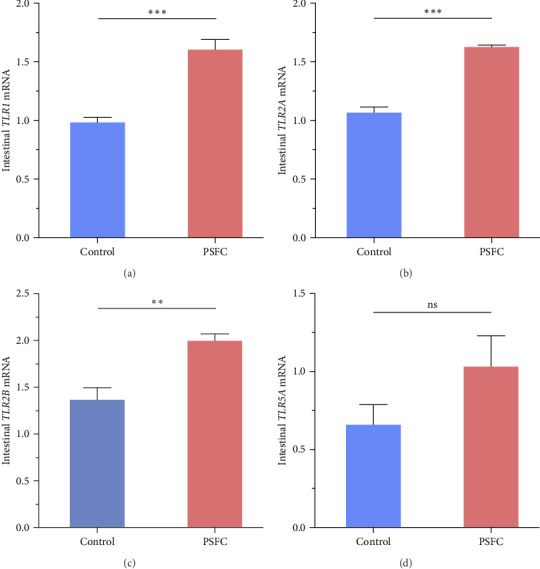
The mRNA levels of *TLR1* (A), *TLR2A* (B), *TLR2B* (C), and TLR5A (D) in the intestines of large yellow croaker (*L. crocea*). Values are presented as mean ± SE, *n* = 4 (*⁣*^ns^*p* > 0.05, *⁣*^*∗*^*p* ≤ 0.05,  ^*∗*^^*∗*^*p* ≤ 0.01,  ^*∗*^^*∗*^*p* ≤ 0.001).

As can be seen from the Figure 6, the expressions of *TLR1* (*p* < 0.001, Figure 6A), *TLR2A* (*p* < 0.001, Figure 6B) and *TLR2B* (*p* < 0.001, Figure 6C) in PSFC group were significantly higher than those in the control group. Intestinal *TLR5A* mRNA level did not vary significantly between control and PSFC groups (*p* > 0 · 05, Figure 6D).

**Figure 7:**
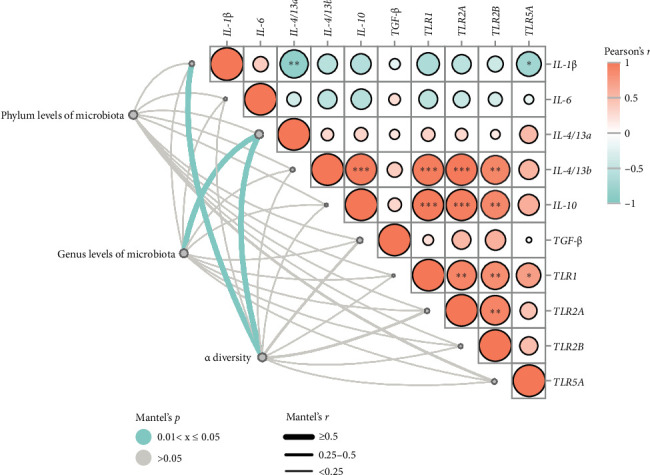
Relationships between bacterial communities and inflammation/TLRs. The correlation heatmap showed that *IL-1*β mRNA level had significant negative correlations with *IL-4/13a* and *TLR5A* mRNA level. There were significantly positive correlations between *TLR1* and *TLR2A*, *TLR2B* and *TLR5A* mRNA levels, as well as *TLR2A* and *TLR2B* mRNA levels. Intestinal *IL-4/13b* and *IL-10* mRNA level had significantly positive correlations with TLR1, TLR2A and *TLR2b* mRNA level (*⁣*^ns^*p* > 0.05, *⁣*^*∗*^*p* ≤ 0.05,  ^*∗*^^*∗*^*p* ≤ 0.01,  ^*∗*^^*∗*^*p* ≤ 0.001).

Pearson's correlation analysis of inflammatory genes and toll-like receptors (TLRs) signaling were shown in Figure 7. The correlation heatmap showed that *IL-1*β mRNA level had significant negative correlations with *IL-4/13a* and *TLR5A* mRNA level (*p* < 0.05). There were significantly positive correlations between *TLR1* and *TLR2A*, *TLR2B* and *TLR5A* mRNA levels, as well as *TLR2A* and *TLR2B* mRNA levels (*p* < 0.05). Intestinal *IL-4/13b* and *IL-10* mRNA level had significantly positive correlations with *TLR1*, *TLR2A* and *TLR2B* mRNA level (*p* < 0.05).

### 3.4. Intestinal Microbiota

**Figure 8:**
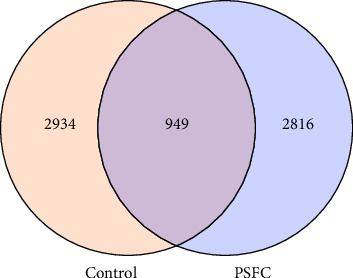
Venn diagram of unique and shared OTUs.

**Figure 9:**
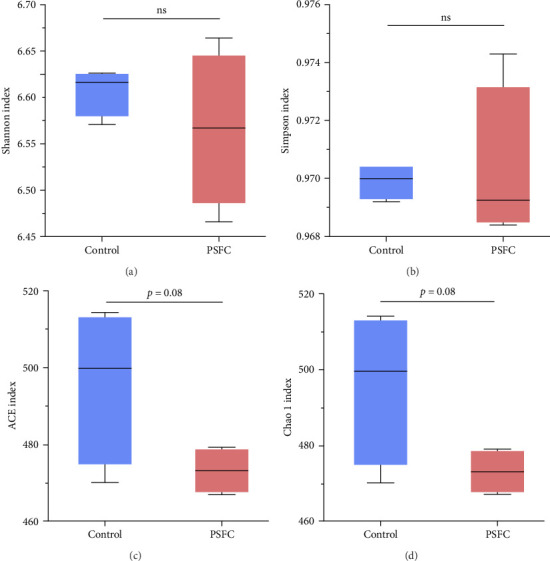
α diversity of intestinal microbiota of large yellow croaker (*L. crocea*). (A) Shannon, (B) Simpson, (C) Ace, and (D) Chao1. Values are presented as mean ± SE, *n* = 4 (*⁣*^ns^*p* > 0.05, *⁣*^*∗*^*p* ≤ 0.05,  ^*∗*^^*∗*^*p* ≤ 0.01,  ^*∗*^^*∗*^*p* ≤ 0.001).

The differences in OTUs were investigated between the control and PSFC groups. A total of 949 OTUs were found to be shared among all samples, while the numbers of unique OTUs were 2934 in the control group, as well as 2816 in the PSFC group (Figure 8). These results indicated that there were diversity differences in the intestinal microbiota of *L. crocea* fed with control and PSFC diets. α diversity was assessed by the indexes of the Ace, Chao1, Shannon, and Simpson. No significant differences of Shannon (Figure 9A) and Simpson (Figure 9B) indexes were observed between the control and PSFC groups (*p* > 0 · 05). Compared to the control groups, Ace (Figure 9C) and Chao1 (Figure 9D) indexes tended to decrease in PSFC group (*p*=0.08). Overall, PSFC resulted in reduced α diversity in the intestinal microbiota of *L. crocea*.

**Figure 10:**
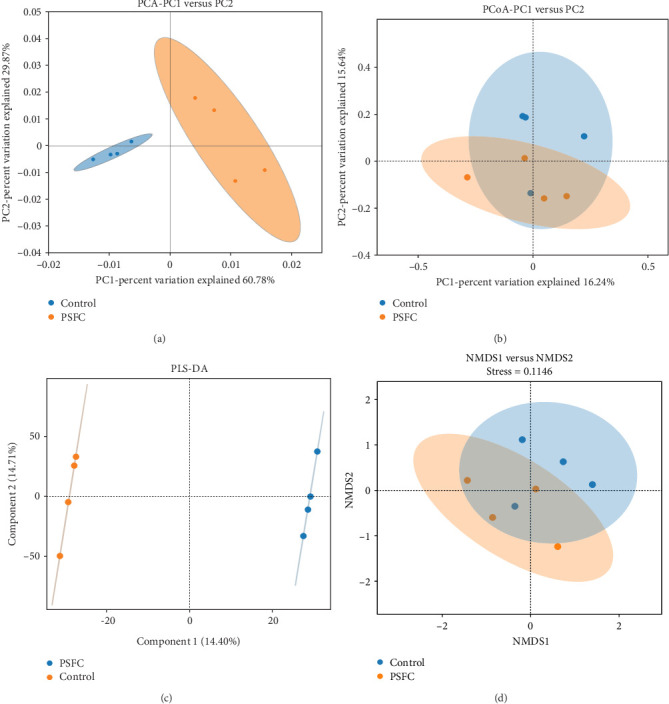
β-diversity of intestinal microbiota of large yellow croaker (*L. crocea*). (A) Principal component analysis (PCA), (B) Principal coordinates analysis (PCoA), (C) Partial least squares discriminant analysis (PLS-DA), and (D) non-metricmulti-dimensional scaling (NMDS).

Figure 10 displayed β-diversity of intestinal microbiota. The samples of two groups were divided into two distinct parts by PCA and PLS-DA, indicating clear variances in microbial composition between the control and PSFC groups. Similarly, apparent differences of microbial composition were found by PCoA and NMDS, although there was a part overlap between the control and PSFC groups.

**Figure 11:**
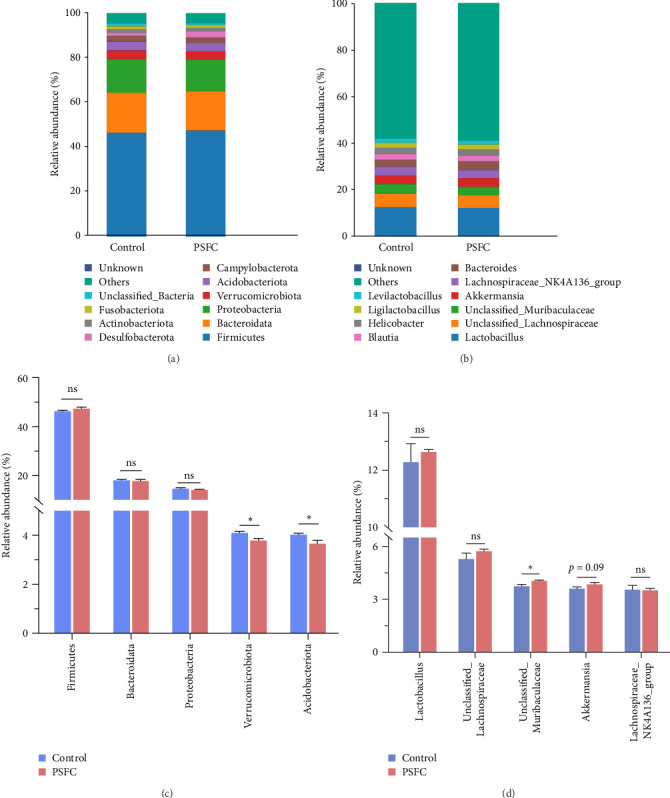
Relative abundances of intestinal microbiota at phyla (A) and genus (B) taxonomic levels. The changes of relative abundances of dominant phyla (C) and genus (D). Values are presented as mean ± SE, *n* = 4 (*⁣*^ns^*p* > 0.05, *⁣*^*∗*^*p* ≤ 0.05,  ^*∗*^^*∗*^*p* ≤ 0.01,  ^*∗*^^*∗*^*p* ≤ 0.001).

The microbial community composition at the phylum level is broadly similar between the control and PSFC groups, with minor but statistically significant differences in some less abundant phyla (e.g., Verrucomicrobiota, Acidobacteriota). The predominant (more than 10%) phyla present in the intestinal microbiota were Firmicutes (46.36% and 47.37%), Bacteroidota (18.09% and 17.19%), and Proteobacteria (14.49% and 14.04%) in the control and PSFC groups (Figure 11A). There were no significant alterations on Firmicutes, Bacteroidota, and Proteobacteria relative abundance between control and PSFC groups (Figure 11C). Compared to the control group, significantly decreased relative abundances of Verrucomicrobiota and Acidobacteriota were observed in PSFC group (*p* < 0.05, Figure 11C). At genus level, *Lactobacillus* (12.28% and 12.63%) was the most predominant in the control and PSFC groups (Figure 11B). At the genus level, most taxa show no significant differences, except for *unclassified_Muribaculaceae* and *Akkermansia* (Figure 11D). Control and PSFC groups had no significant relative abundances of *Lactobacillus*, *unclassified_Lachnospiraceae*, and *Lachnospiraceae_NK4A136_group*. Compared to the control group, *unclassified_Muribaculaceae* significantly increased in PSFC group (*p* < 0.05), as well as an increased tendency on *Akkermansia* relative abundance (*p*=0.09).

To investigate relationships between intestinal microbiota and inflammatory genes/TLRs signaling (Figure 7), we used Mantel tests. Phylum levels of intestinal microbiota showed a tendency to be significantly correlated with *TLR5A* mRNA level (*p*=0.051). Genus levels of intestinal microbiota had correlations with *IL-4/13a* mRNA level (*p* < 0.05), as well as *IL-4/13b* (*p*=0.051). The Mantel test revealed significant relationships between alpha diversity and *IL-1*β and *IL-4/13a* mRNA levels (*p* < 0.05). Furthermore, alpha diversity tended to be correlated with *TGF*-β(*p*=0.08) and *TLR2A*(*p*=0.09) mRNA levels.

**Figure 12:**
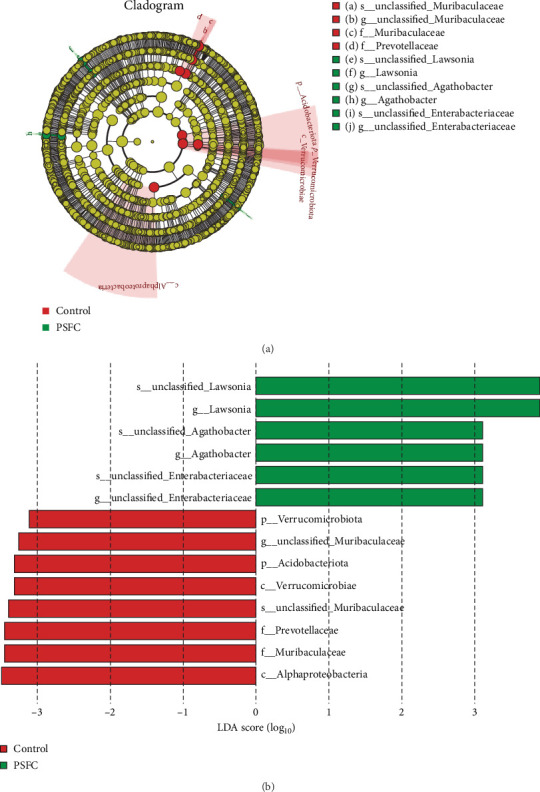
The difference of intestinal microbiota of large yellow croaker (*L. crocea*). (A) Linear discriminant analysis effect size (LEfSe) cladogram, and (B) Linear discriminate analysis (LDA) score.

Linear discriminant analysis (LDA) effect size (LEfSe) analyses used to determine features most likely to explain differences between the control and PSFC groups were presented in Figure 12. LEfSe showed Verrucomicrobiota and Acidobacteriota enrichment in the control group (LDA > 3, *p* < 0.05). At the genus level, LEfSe analysis revealed that *Lawsonia*, *Agathobacter*, and *unclassified_Enterobacteriaceae* were significantly enriched in the PSFC group but not in the control group (LDA > 3, *p* < 0.05).

**Figure 13:**
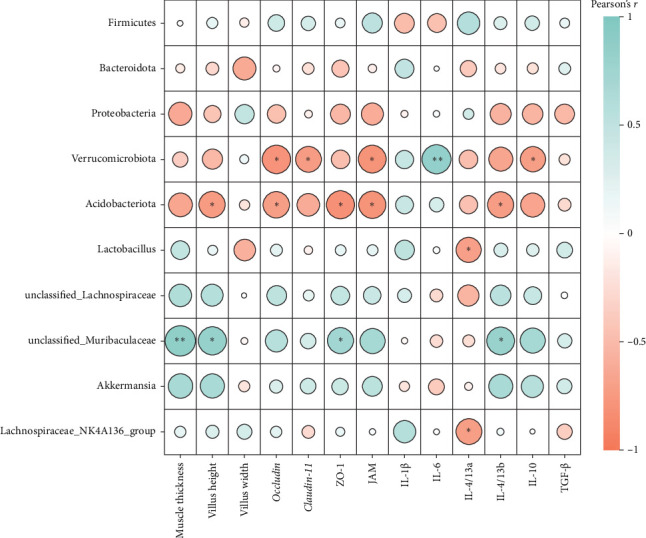
Correlations between relative abundances of intestinal microbiota and the intestinal health status (including morphometric parameters, inflammatory and tight junction-related genes expression). The correlation heatmap showed that Verrucomicrobiota abundance had significant negative correlations with *occludin*, *claudin-11*, *JAM*, and *IL-10* mRNA level. There were significantly negative correlations between Acidobacteriota abundance and villus height, *occludin*, *ZO-1*, *JAM*, and *IL-4/13b* mRNA levels. Intestinal *unclassified_Muribaculaceae* abundance had significantly positive correlations with muscle thickness, villus height, *ZO-1*, and *IL-4/13b* mRNA levels (*⁣*^ns^*p* > 0.05, *⁣*^*∗*^*p* ≤ 0.05,  ^*∗*^^*∗*^*p* ≤ 0.01,  ^*∗*^^*∗*^*p* ≤ 0.001).

Pearson's correlation analysis of intestinal microbiota community and intestinal histology, tight junctions, as well as inflammatory genes were displayed in Figure 13. The correlation heatmap showed that Verrucomicrobiota abundance had significant negative correlations with *occludin*, *claudin-11*, *JAM*, and *IL-10* mRNA level (*p* < 0.05). There were significantly negative correlations between Acidobacteriota abundance and villus height, *occludin*, *ZO-1*, *JAM*, and *IL-4/13b* mRNA levels (*p* < 0.05). Intestinal *unclassified_Muribaculaceae* abundance had significantly positive correlations with muscle thickness, villus height, *ZO-1*, and *IL-4/13b* mRNA levels (*p* < 0.05).

## 4. Discussion

Microbial fermentation process is beneficial to improve flavor and nutritional value of functional feeds, such as increased content of small-sized peptides and eliminate various antinutritional factors [21]. It has been reported that turbot (*S. maximus* L.) fed *E. faecium* fermented SBM showed more superior growth performance than that fed SBM [17]. In line with the previous study, dietary PSFC significantly enhanced WGR and SGR compared to the control group in the present study, indicating improved growth performance. The growth promoting role for PSFC could attribute to antinutritional factors degradation and bioactive compounds (naringin, essential oils, pectin, polyphenols, etc.) in pomelo peel, which are beneficial for intestinal health. Thus, the intestinal health status will be further discussed in *L. crocea* fed with two different diets.

The intestinal barrier plays a crucial role in maintaining the intestinal and even systemic development and health of aquatic animals [22]. Intestinal epithelium acts as a physical barrier that allows nutrients to be absorbed while defensing against pathogens [20, 23] therefore an intact intestinal mucosal barrier is important for proper function of intestine. DAO and *D*-lactate can serve as two markers of intestinal permeability. It has been reported that DAO activity and *D*-lactate concentration in serum were increased when the integrity of intestinal barrier was broken [24–26]. In the present study, serum DAO activity and *D*-lactate concentration decreased in the PSFC group, implying superior intestinal integrity and barrier function. These results coincide with the intestinal histological observation, which was characterized with increased muscle thickness and villus height in the PSFC group. A possible explanation for these results might be that microbial fermentation can improve nutrient availability and bioavailability, increased content of small-sized peptides and eliminate various antinutritional factors in SBM, making them more easily digestible which eventually improve growth and health performance of fish [11]. It has been reported that the replacement of SBM by fermented SBM improved intestinal histology of largemouth bass (*M. salmoides*), characterized with higher villus height, villus width and muscular thickness [12]. Similarly, turbot (*S. maximus* L.) fed *E. faecium* fermented SBM had higher villus and microvillus height and fewer inflammatory cell infiltration than that fed SBM, indicated improvement of intestinal histology [17]. Another possible alternative explanation of our findings is that pomelo peel is rich in various nutrients and functional compounds (naringin, essential oils, pectin, polyphenols, etc.), which is beneficial for intestinal and systemic health [1, 2]. For example, naringin reduced dysplastic cells and improved structure and nuclei of epithelium in small and colonic intestines of adenomatous polyposis coli multiple intestinal neoplasia (*Apc*^*Min*/+^) mice [4]. This result is consistent with other research which showed naringin mitigated dextran sulfate sodium (DSS)-induced disease activities index (DAI), colon length shortening, and colonic histological injury in mice [3, 27].

Current evidences suggest that stimulation of tight junction proteins (*occludin*, *claudins*, *JAMs*, *ZOs*, etc.) is conducive to maintaining intestinal integrity and reduced permeability [20, 28, 29]. For instance, it has been reported that naringin intervention promoted *ZO-1* and *occludin* expressions, therefore alleviating DSS-induced colonic histopathological injury in mice [3, 27]. A similar situation also prevailed in aquatic animals that upregulation of tight junction proteins was generally accompanied by superior intestinal histology in spotted seabass (*L. maculatus*) [20], largemouth bass (*M. salmoides*) [30], as well as common carp (*Cyprinus carpio*) [31]. Consistent with the previous studies, dietary PSFC upregulated the expressions of *occludin*, *claudin-11*, *ZO-1*, and *JAM*, and Pearson's correlation analysis revealed that intestinal histology had positive correlations with tight junction related genes (especially *ZO-1* and *JAM*) mRNA levels in this study. Taken together, these results suggest that the upregulation of tight junction proteins could be attributed to microbial fermentation and functional compounds in pomelo peel, and eventually improve intestinal barrier integrity and superior histology.

In the intestine, modulation of inflammatory response is strongly related to the normal mucosal barrier functions. There is a growing body of evidence to reveal that the inhibition of intestinal inflammation can effectively mitigate intestinal mucosal barrier injury, regardless of mice and aquatic animals [3, 13, 20, 30, 32]. It is important to maintain a dynamic equilibrium between proinflammatory and anti-inflammatory cytokines in the body. Overproduction of proinflammatory cytokines and/or underproduction of anti-inflammatory cytokines can result in inflammatory disturbance, which can induce damage to organs and tissues [13, 33]. Therefore, it is crucial to keep these processes in check and ensure that the body's immune response is functioning optimally. High percentage of dietary SBM could induce enteritis in *L. crocea*, showing increased proinflammatory cytokine production (*IL-1*β, *IL-6*, and *TNF*-α) and decreased anti-inflammatory cytokine expression (*IL-4/13a*, *IL-4/13b*, and *TGF*-β) [14, 15]. This may be due to the high content of antinutritional factors in SBM. Emerging evidence also suggests that soy glycinin, a major antinutritional factors in SBM, can cause intestinal inflammation by activating the MAPK/NF-kappa B signaling route in *L. crocea* [16]. Many functional compounds in pomelo peel, such as naringin and essential oils, present excellent anti-inflammatory function [2, 34]. Recent researches have reported that naringin inhibited DSS-induced *IL-1*β, *IL-6* and tumor necrosis factor-α (*TNF*-α) expressions in mice colon tissue [3, 27]. In addition, microbial fermentation attenuated SBM-induced intestinal inflammation in turbot (*S. maximus* L.) by downregulating proinflammatory *IL-1*β, *TNF*-α, *IL-8*, interferon-γ and *IL-22* expressions [17, 35]. The microbial fermentation of SBM can greatly degrade antinutritional factors that can result in excessive inflammatory response[11, 21 ]. In this study, inhibition of proinflammatory *IL-1*β and promotion of anti-inflammatory *IL-4/13b* and *IL-10* expressions were documented in PSFC group. These findings indicated that PSFC is helpful in relieving intestinal inflammation of *L. crocea*, which is consistent with the results of intestinal histology.

TLRs, as a family of pattern recognition receptors, can initiate innate immune responses [36, 37]. Previous evidence suggests that TLRs signaling is a critical factor in keeping intestinal homeostasis responses to detrimental factors by modulating the inflammatory and histological repair [20, 38]. A previous study has showed that activation of TLRs signaling (including *TLR1*, *TLR2*, *TLR3*, and *TLR5*) can induce synthesis and secretion of antimicrobial peptides, a type of vital component of innate immunity in fish [37]. In this study, dietary PSFC upregulated intestinal *TLR1*, *TLR2A*, and *TLR2B* expression, which had significantly positive correlations with improved inflammatory status. This result may be explained by the emerging evidences that flavonoid extract, volatile oil, and naringin from pomelo peel could alleviate inflammation via regulating TLRs signaling pathway [4, 18, 39]. Another possible explanation for this is that probiotics in PSFC could lead to activation of intestinal TLRs signaling, which is in line with previous studies [20, 24, 40].

The capability of the intestinal microbiota to modulate barrier functions has become a crucial aspect of host-microbe interaction in the intestine [22, 29, 40]. In this study, we found that the modulation of inflammatory response and TLRs signaling had correlations with intestinal microbiota. In addition, PSFC application caused clear alterations in the intestinal microbiota of *L. crocea*, which characterized with decreased relative abundances of Verrucomicrobiota and Acidobacteriota and increased relative abundances of *unclassified_Muribaculaceae* and *Akkermansia*. Overall, the microbial community structure remained largely consistent across the two groups, with most taxa showing no significant divergence. However, the observed shifts in Verrucomicrobiota, Acidobacteriota, and *unclassified_Muribaculaceae* highlight potential functional implications of PSFC intervention, warranting further investigation into their roles in gut microbiota dynamics. The near-significant trends in other genera (*Akkermansia* with *p*=0.09) also suggest the possibility of subtle but biologically relevant effects that may require further study. Recent evidence suggests that *Muribaculaceae* are versatile concerning complex carbohydrate degradation [41, 42]. The genus *Akkermansia* has been shown numerous beneficial functions and the increased abundance of this commensal bacterium can improved mucosal and systemic health [43]. It has been found in turbot (*S. maximus*) that dietary oregano oil and probiotics supplementation improved growth performance and immunity by affecting intestinal microbiota (enhanced the relative abundances of potential beneficial bacteria, such as *Akkermansia*, *Bifidobacteium*, and *Faecalibacterium*) [44]. Herein, we speculated that modulation of intestinal microbiota could be attributed to functional compounds (such as naringin and essential oils in pomelo peel) and probiotics in dietary PSFC, eventually improving intestinal barrier functions. However, *B. pumilus* SE5, *S. cerevisiae* Sa and *L. lactis* 17 were not detected in the intestinal microbiota in this study. It is possible that the drying process following fermentation could have affected the activity and detectability of these bacteria strains. High temperatures or other drying conditions might have reduced the viability or altered the genetic material of the probiotics, making them difficult to identify in the gut flora analysis. Moreover, the methods used for intestinal microbiota analysis (e.g., 16S rRNA gene sequencing in this study) may have limitations in detecting certain bacterial genera or species. The primers used in the sequencing process might not have been optimal for amplifying the DNA of these bacteria strains, leading to underrepresentation or complete absence of these bacteria in the results. Furthermore, pearson's correlation analysis showed that phylum Verrucomicrobiota and Acidobacteriota and genus *unclassified_Muribaculaceae* might as key microbiota in improving intestinal barrier functions, including intestinal histology, tight junction proteins, as well as inflammatory response. Interestingly, we found that *L. crocea* fed with PSFC exhibited a distinct pomelo-like aroma during sampling. However, the potential of PSFC to enhance flesh quality and flavor profile, as well as the underlying mechanisms governing flavor compound deposition in fish tissues, remain unclear and require systematic investigation.

## 5. Conclusion

In this investigation, the aim was to assess the potential role of PSFC in *L. crocea*. The findings clearly indicate that dietary PSFC improved weight gain and intestinal histology, positively regulated inflammatory response, and TLRs signaling, as well as reshaped intestinal microbiota homeostasis. Overall, PSFC can significantly improve growth and intestinal health of *L. crocea*, and be used as a suitable protein source on *L. crocea* culture. This work offers a novel understanding of the pomelo peel, which is beneficial for mitigating environmental pollution and reducing the cost of aquaculture.

## Figures and Tables

**Table 1:** Formulation and proximate composition of the experimental diets (%).

Ingredients	Control	PSFC
Fish meal^a^	30.00	30.00
Chicken meal^a^	10.00	10.00
Soybean meal (SBM)^a^	11.00	0
Pomelo peel/SBM fermented compound^a^	0	11.00
Wheat starch^a^	25.00	25.00
Corn gluten meal^a^	4.00	4.00
Soybean protein concentrate^a^	8.00	8.00
Soybean oil	1.50	1.50
Fish oil	2.50	2.50
Lecithin	1.00	1.00
Stickwater	4.50	4.50
Vitamin C	0.10	0.10
Vitamin premix^b^	0.40	0.40
Mineral premix^c^	0.50	0.50
Calcium dihydrogen phosphate	1.00	1.00
Choline chloride	0.50	0.50
Proximate analysis (%)	—	—
Crude protein	44.16	44.32
Crude lipid	10.56	10.57
Lysine	2.04	1.97
Methionine	0.73	0.73
Total *p*	1.26	1.34

^a^Supplied by Xiamen Jiakang Aquatic Feed Co., Ltd. (Fujian, China). Chicken meal, 65.44% crude protein and 9.28% crude lipid; Corn gluten meal, 62.37% crude protein and 2.20% crude lipid; Fish meal, 63.2% crude protein and 10.27% crude lipid; Pomelo peel/SBM fermented compound, 47.76% crude protein and 1.10% crude lipid; Soybean meal, 47.40% crude protein and 1.50% crude lipid; Soybean protein concentrate, crude protein 67% and 0.6% crude lipid; Wheat flour, 16% crude protein and 1.5% crude lipid.

^b^Vitamin premix containing the following (mg/kg diet): alpha-tocopherol, 100; biotin, 2; cholecalciferol, 5; ethoxyquin, 150; folic acid, 2; inositol, 100; niacin acid, 50; pantothenic acid, 20; pyridoxine HCl, 10; retinol acetate, 400; riboflavin, 8; thiamin, 10; vitamin B12, 0.2; vitamin K3, 10; wheat middling 132.8.

^c^Mineral premix containing the following (mg/kg diet): CoSO_4_, 100; CuSO_4_ · 5H_2_O, 24; FeSO_4_ · H_2_O, 400; KCI, 200; KI, 60; MgSO_4_ · 7H_2_O, 800; MnSO_4_ · H_2_O, 78; Na_2_SeO_3_, 50; Zeolite, 311.4; ZnSO_4_ · H_2_O, 174.

**Table 2:** Growth performance, feed utilization, and survival rate.

Item	Control	PSFC	*p*-Value
FBW (g)	49.24 ± 2.44	54.32 ± 2.80	0.086
WGR (%)	154.51 ± 19.74^b^	178.00 ± 25.39^a^	0.015
SGR (%/day)	2.22 ± 0.19^b^	2.42 ± 0.22^a^	0.036
FCR	1.14 ± 0.33	1.09 ± 0.32	0.173
CF (g/cm^3^)	1.66 ± 0.17	1.82 ± 0.32	0.163
VSI (%)	6.66 ± 1.47	6.55 ± 0.2.11	0.430
HSI (%)	1.31 ± 0.49	1.52 ± 0.73	0.084
SR (%)	95.50 ± 3.70	92.25 ± 2.06	0.076

*Note:* Different lowercase letters of shoulder label indicate significant difference (*p* < 0.005).

## Data Availability

Data that support the findings of this study are available from the corresponding author upon reasonable request.

## Supporting Information

Supporting Information Table S1: Primers used for real-time quantitative PCR.
