# Supplementary material for: Pomelo Peel and Soybean Meal Fermented Compound as Feedstuff for Large Yellow Croaker (Larimichthys crocea): A Study on Growth and Intestinal Health
Source: Aquac Nutr. 2025 Jul 11;2025:6556868. doi: 10.1155/anu/6556868 (PMC12274105; doi:10.1155/anu/6556868)
Supplement: Supporting Information — Table S1: Primers used for real-time quantitative PCR. [file 6556868.f1.docx]

**Supplementary Table S1** Primers used for real-time quantitative PCR.

| Genes | Forward primers (5’→3’) | Reverse primers (5'-3') |
| --- | --- | --- |
| *β-actin* | GACCTGACAGACTACCTCATG | AGTTGAAGGTGGTCTCGTGGA |
| *il-1β* | CAGCTGTTCTCAAGTATGTGGC | GTTGTAAATAGTGGGTGTGTCG |
| *il-4/13a* | TGGTACTGCTGGTCAATCCG | TTTTGCCTTCAGCCAGATGT |
| *il-4/13b* | AGTTCTTCTGTCGCGCTGAG | GCTATGTATGTGCGGTTGCTG |
| *il-6* | GCTGTTCTCAAGTATGTGGCG | TGTTGTAAATAGTGGGTGTGTCG |
| *il-10* | AGTCGGTTACTTTCTGTGGTG | TGTATGACGCAATATGGTCTG |
| *tgf-β* | AGCAACCACCGTACATCCTG | AGGTATCCCGTTGGCTTGTG |
| *occludin* | AGGCTACGGCAACAGTTATG | GTGGGTCCACAAAGCAGTAA |
| *claudin-11* | ACCTCCGCCATCAAGCA | TGGGACAAAGAGCCACATC |
| *zo-1* | TGTCAAGTCCCGCAAAAATG | CAACTTGCCCTTTGACCTCT |
| *jam* | CAAGACTGTGTGGGCAAACG | CACATAACTGGGGATGCCGT |
| *tlr1* | CTTTGTCAAGAGCGAGTGGT | GGTTCATCATGGCCTTCAGC |
| *tlr2a* | GTCCGACAACCTGCTGACTGA | CAGGTGGGTGAGTTTGGAGAG |
| *tlr2b* | ATGATGTGCTATGGCGAGGG | TCGGCAAACATGTGGTCACT |
| *tlr5a* | GGCACAGTGAGGAAAGGT | TAGCAAGCGTCCACATAC |
